# Primary Effusion Lymphoma Secondary to Human Herpesvirus 8 (HHV-8) Infection in an Immunocompetent Host: A Case Report

**DOI:** 10.7759/cureus.55774

**Published:** 2024-03-08

**Authors:** Sagar Pandey, Ernestine Faye S Tan, Myo Myint Tun, Amulya Bellamkonda, Shwe Yee Htet, Kalendra Kunwar, Madhumati Kalavar

**Affiliations:** 1 Internal Medicine, One Brooklyn Health/Interfaith Medical Center, Brooklyn, USA; 2 Internal Medicine, NewYork-Presbyterian Westchester Hospital, Bronxville, USA; 3 Hematology and Oncology, One Brooklyn Health/Interfaith Medical Center, Brooklyn, USA

**Keywords:** non-hodgins lymphoma, lymphoma, hhv 8, immunocompetent patients, primary effusion lymphoma

## Abstract

Primary effusion lymphoma (PEL) is a rare, aggressive, mature type of B-cell lymphoma that usually causes malignant, lymphomatous effusions in the absence of a solid mass. This is commonly seen in immunosuppressed individuals such as those with underlying malignancies, human immunodeficiency virus infection (HIV), cirrhosis, and a history of solid organ transplantation who are infected with human herpesvirus 8 (HHV-8). Clinical presentation varies depending on the extent of disease like shortness of breath, abdominal distention, and typical B symptoms like weight loss, fever, and night sweats. Morphological and immunohistochemical analysis of pleural fluid is required for diagnosis of PEL. Recent case studies are increasingly being reported with cases of PEL presenting in immunocompetent individuals infected with HHV-8. We present a case of PEL in an immunocompetent host and highlight its presentation, diagnosis, and management approaches. Due to the well-known association of PEL with immunocompromised status, the diagnosis is often overlooked in immunocompetent individuals. This case would further highlight the increasing association and the need for clinical vigilance in diagnosing PEL in immunocompetent patients.

## Introduction

Primary effusion lymphoma (PEL) is a rare, aggressive, high-grade type of non-Hodgkin’s lymphoma (NHL) that accounts for <1% of B-cell lymphomas and about 4% of the human immunodeficiency virus (HIV) associated lymphomas [1,2]. However, rare cases of PEL have been reported in HIV-negative individuals in the form of isolated case reports [3,4]. Its unique characteristic is its predilection for body cavities such as pleural spaces, the pericardial cavity, and the peritoneum, where it commonly forms malignant lymphomatous effusions usually in the absence of a discrete mass [1,2]. PEL, previously termed body cavity lymphoma, was first reported in the late 1980s to 1990s as an acquired immunodeficiency syndrome (AIDS)-associated type of NHL due to its high prevalence in immunocompromised patients in the advanced stages of AIDS [5,6]. The causative agent is a gamma 2 herpes virus, the human herpesvirus 8 (HHV-8), otherwise termed a Kaposi-sarcoma-associated herpes virus (KSHV) [7]. Further studies also found that 60-90% of these infections were co-infected with Epstein-Barr virus (EBV), which similarly causes AIDS-associated malignancies [8,9]. 

However, recent evidence has emerged regarding rare cases of PEL in immunocompetent, HIV-negative, individuals infected with HHV-8 [8]. We hereby present a case of primary effusion lymphoma in an immunocompetent host to shed light on the condition and share clinical insights on presentation, diagnosis, and approach to management of the condition. This case would further elucidate the diagnosis of PEL in the absence of immunocompromised status and thereby highlight the importance of clinical vigilance to prevent missing out on the diagnosis of PEL in immunocompetent patients.

## Case presentation

A 76-year-old male with a past medical history of hypertension, type 2 diabetes mellitus, chronic kidney disease stage 3b, stage 2A gastric adenocarcinoma, and hyperlipidemia presented to the emergency department with shortness of breath and palpitations for the past three days. The patient reported a racing of the heart along with shortness of breath on minimal exertion. The patient denied any history of palpitations in the past, along with a negative history of fever, cough, chest pain, and light-headedness. The patient denied any history of blood in stool, passage of black tarry sticky stool, diarrhea, recent significant weight loss, or any thyroidal illness in the past. 

Significant past medical history included a history of stage 2A gastric adenocarcinoma status post D2 gastrectomy and chemoradiotherapy. Yearly surveillance with esophagogastroduodenoscopy (EGD) with gastric stump biopsy was negative for malignancy. The patient also had a history of recurrent admissions with symptomatic anemia. Workup for anemia was negative for multiple myeloma, myelodysplastic syndrome, or leukemia. Iron studies were suggestive of anemia of chronic disease with a suspected component of gastrointestinal bleeding (negative EGD/colonoscopy but suspected arteriovenous malformation on red cell tagged nuclear scan). 

At presentation, the patient was awake, alert, oriented to time, place, and person, afebrile, tachycardic, and with an oxygen saturation of 98% on room air. Vitals signs were within normal range apart from the resting tachycardia. A cardiovascular exam revealed irregularly irregular rhythm and tachycardia, distant heart sounds, and no audible murmurs. Bilateral decreased air entry with scattered crackles was auscultated on a pulmonary exam with normal respiratory rate and effort. On an abdominal exam, residual well-healed scarring from previous abdominal surgery was visible. The abdomen was soft and non-tender with normal bowel sounds. 

**Figure 1:**
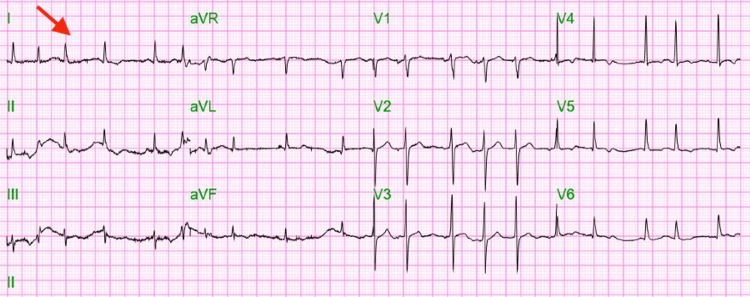
Electrocardiogram at presentation depicting atrial fibrillation with rapid ventricular rate

**Figure 2:**
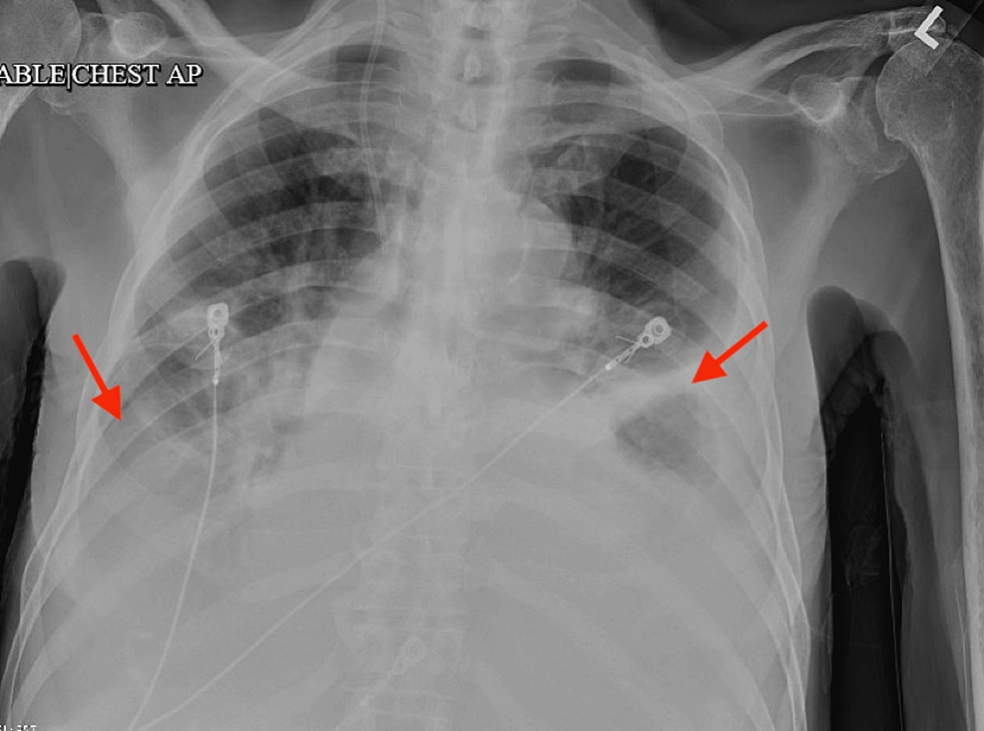
Chest X-ray at presentation showing bilateral pleural effusion (left > right) with associated atelectasis/consolidation

**Figure 3:**
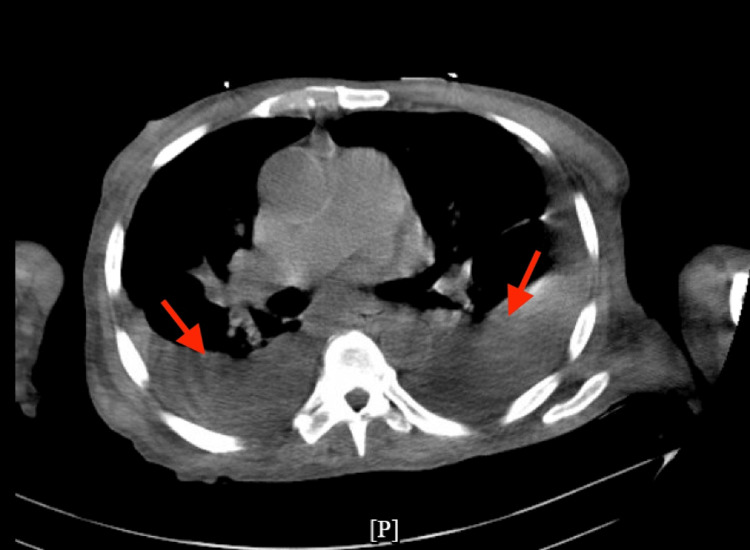
CT chest without contrast with bilateral pleural effusion with atelectasis

Twelve lead electrocardiograms (EKG) revealed atrial fibrillation with a rapid ventricular rate (heart rate: 133) which was subsequently normalized with intravenous followed by daily oral diazepam (Figure 1). Chest X-ray (CXR) showed bilateral pleural effusion (left > right) with associated atelectasis/consolidation (Figure 2). Computed tomography (CT) scan chest without contrast subsequently confirmed bilateral large pleural effusion with atelectasis (Figure 3).

**Figure 4:**
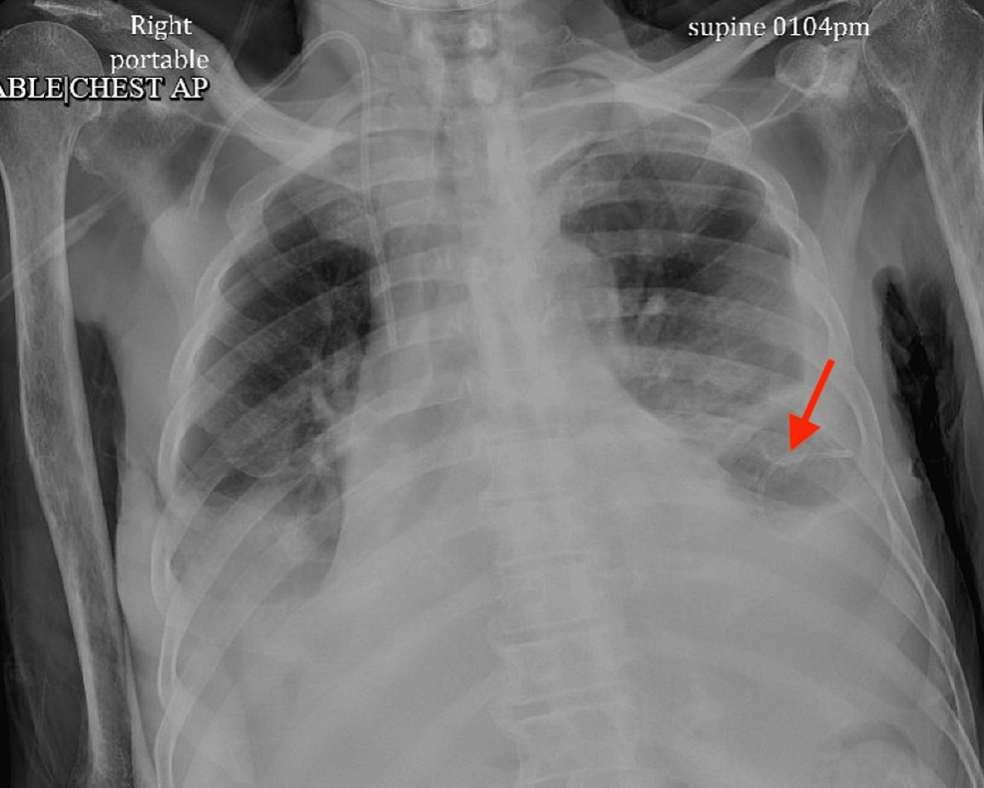
Chest X-ray post placement of left-sided pigtail thoracentesis catheter showing significant reduction of left pleural effusion

Left thoracentesis and placement of 6F pigtail thoracentesis catheter was done with the removal of one liter of bloody fluid after consultation with the pulmonary team. Pleural fluid analysis and cytology were sent. The patient was placed on the nasal cannula as he was desaturating to 87% on room air. Post-procedure CXR showed a significant reduction of left pleural effusion compared to previous studies (Figure 4).

Blood cultures sent at admission were reported as negative. However, sputum culture was positive for moderate *Enterococcus* and extended-spectrum β-lactamase (ESBL) *Klebsiella*. Intravenous antibiotics were switched to a renally adjusted dose of meropenem (ceftriaxone and azithromycin were discontinued) upon consultation with the infectious disease team. An echo done to assess cardiac function reported a normal ejection fraction (55-60 %) with grade one diastolic dysfunction. The patient underwent multiple units of packed red blood cell transfusions due to borderline low hemoglobin and in preparation for the procedure. Laboratory parameters at the presentation are shown in Table 1.

**Table 1:** Laboratory parameters at presentation eGFR: estimated glomerular filtration rate, AST: aspartate transaminase, ALT: alanine transaminase, ALP: alkaline phosphatase, PT: prothrombin time, INR: international normalized ratio.

Lab parameters	Admission values	Normal range
Hemoglobin	7.9	13-17 gm/dL
Hematocrit	24.8	39-53%
White Blood Cells	7.4	4.5-11 10x3/uL
Platelets	82	130-400 10x3/uL
Blood urea nitrogen	38.5	8.4-25.7 mg/dL
Creatinine	2.10	0.72-1.25 mg/dL
eGFR	32	Greater than or equal to 90 ml/min/1.73m2
Sodium	142	136-145 mEq/L
Potassium	3.9	3.5-5.1 mEq/L
AST	16	5-34 U/L
ALT	<10	10-55U/L
ALP	124.6	40-150 U/L
Total protein	7.4	6-8.3 g/dL
Albumin	2.2	3.2-4.6 g/dL
Total bilirubin	0.8	0.2-1.2 mg/dL
PT	13.7	9.8-13.4 secs
INR	1.20	0.85-1.15
Lactic acid	1.6	0.5-1.9 mmol/L

**Figure 5:**
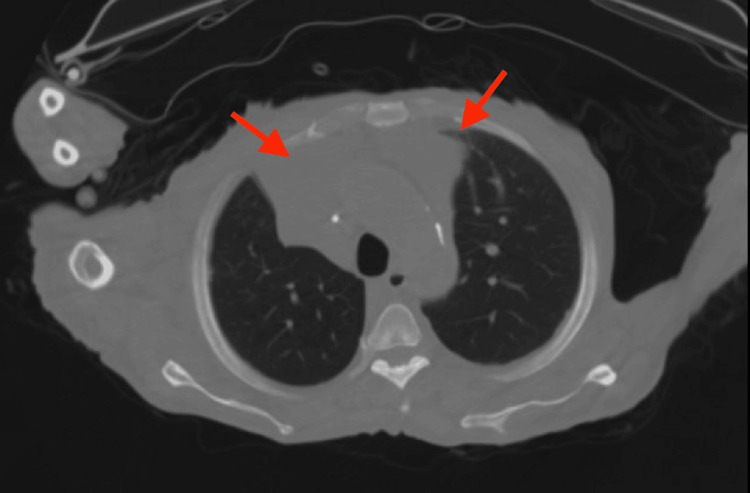
Repeat CT chest with new loculated fluid along bilateral upper mediastinum with moderate to large pleural effusion

CT chest repeated after day 5 of left-sided pigtail catheter placement revealed new loculated fluid along bilateral upper mediastinum with moderate to large pleural effusion (greater on the right side with left pleural effusion decreased in size post pigtail catheter placement) as shown in Figure 5.

**Figure 6:**
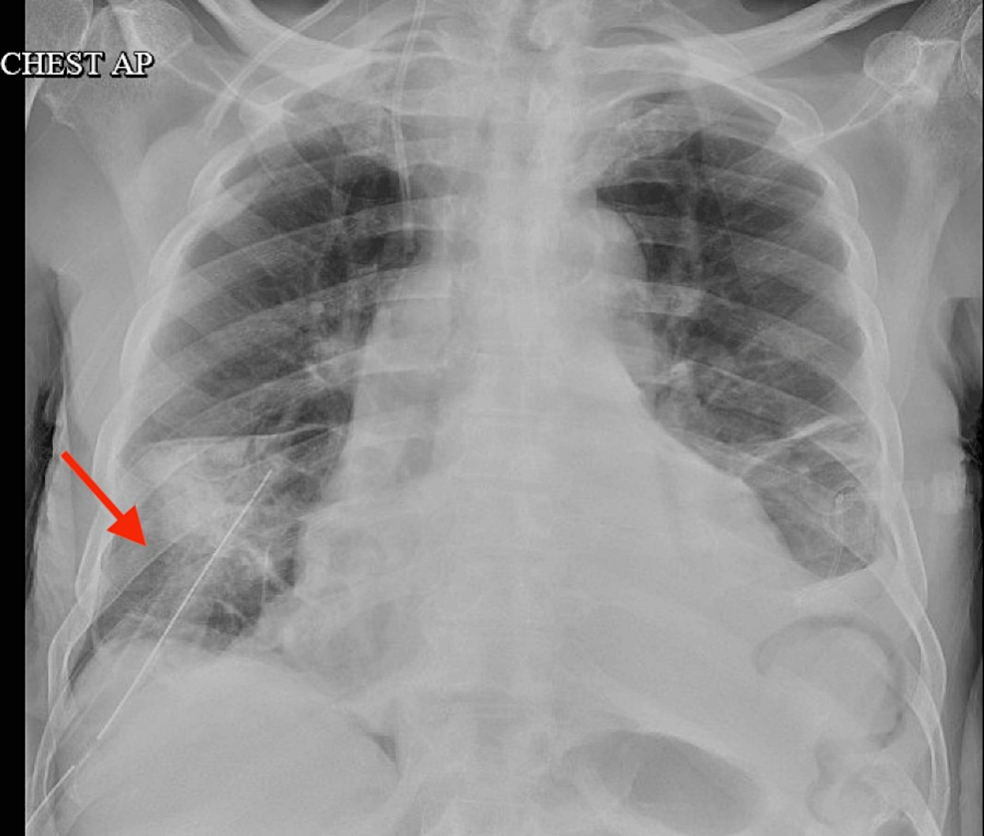
Chest X-ray after placement of right-sided chest tube showing significant improvement of right-sided pleural effusion

An additional 500 ml of pleural fluid was drained from the left pigtail catheter on day 6 of its placement. Cardiothoracic surgery was consulted, a new right-sided chest tube was placed and doxycycline chemical pleurodesis was done (with 500 mg doxycycline in 50 ml of 1% lidocaine, a total of three times on subsequent days). About 1500 ml of serous fluid was drained from the right side. Post-procedure CXR showed improvement in right-sided pleural effusion and trace left pleural effusion (Figure 6).

**Figure 7:**
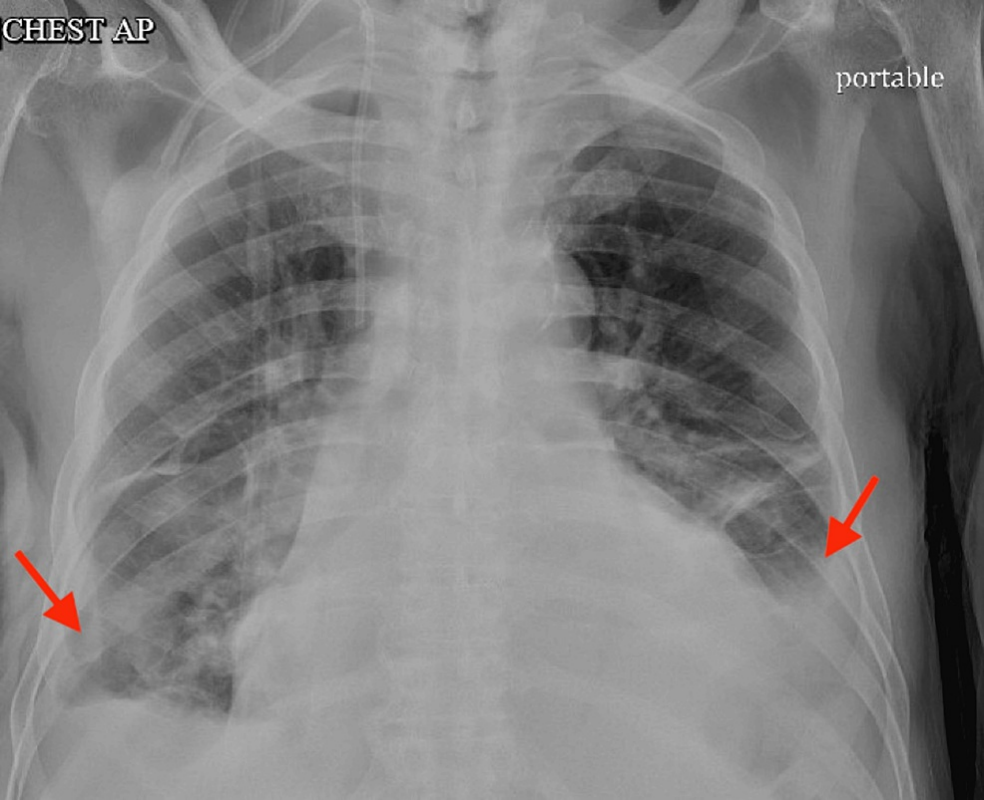
Chest X-ray after removal of chest tube showing small left pleural effusion with interval improvement on right side

Left-sided pigtail catheter was removed on day 12 of placement as the drainage was <100 ml in 24 hrs. The right-sided chest tube was removed on day 9 of placement. Repeat CXR showed small left pleural effusion with interval improvement on the right side (Figure 7).

Pleural fluid cell count with differential revealed hemorrhagic lymphocytic predominant leukocytosis (white blood cells: 3000 with neutrophil: 10.5%, lymphocyte: 84.2%, monocyte: 5.3%). Pleural fluid analysis met the criteria for exudative effusion (pleural fluid protein: 5.4 g/dL, serum protein: 7.2 g/dL, pleural fluid lactate dehydrogenase: 3880 IU/L, serum lactate dehydrogenase: 340 IU/L) with negative acid-fast bacilli smear and culture. The immunohistochemical analysis reported cell block showing atypical large lymphoid cells positive for cluster of differentiation 45 (CD45), CD43, CD30, BOB-1, and HHV-8. EBV-encoded small RNAs (EBER), CD20, CD79a, CD3, CD5, and other T-cell markers were negative. Findings were thus consistent with a primary effusion lymphoma. CDX2, calretinin, CEA, CK7, CD20, AE1/AE3, MUC5AC, SATB2 were negative. Test for HIV along with hepatitis panel was negative. To note, the results for pleural fluid analysis were negative for malignant cells in the first sample of pleural fluid analysis and positive in the second sample. The patient signed out of the hospital against medical advice to follow up in the oncology clinic on an outpatient basis. Bone marrow biopsy was done in the hematology clinic which did not show any bone marrow involvement (no clonal B-cell gene rearrangement, no evidence of acute leukemia or increased blasts, no evidence of abnormal myeloid population, no evidence of atypical T-cell by flow cytometry).

The patient was started on cyclophosphamide, doxorubicin, vincristine, and prednisone (CHOP) chemotherapy regimen with granulocyte colony-stimulating factor (G-CSF) support. Positron emission tomography-computed tomography (PET-CT) scan reported no findings suggestive of recurrent disease in the gastrointestinal tract, no definite abnormal findings to suggest active or progressing nodal, splenic, or marrow sites of lymphoproliferative disease, and no hypermetabolic activity.

## Discussion

PEL primarily affects the male population (male: female ratio of 6:1) with a median age of diagnosis of 45 years old [9,10]. Cases are primarily seen in immunosuppressed individuals, such as those with malignancies, HIV, cirrhosis, or a history of solid organ transplants, although rare cases have been reported in immunocompetent, HIV-negative patients [9]. The mechanism of oncogenesis remains inadequately explored. It is proposed that HHV-8 affects B-cells and enters a latent phase, where many viral transcripts are expressed to promote oncogenesis [9]. One of these includes the latency-associated nuclear antigen (LANA), which is proposed to repress the tumor suppressor protein p53 and the retinoblastoma protein, resulting in tumor survival and proliferation [7,11]. Another culprit is the viral FLICE-like inhibitory protein (FLIP), which is known to activate nuclear factor kappa B (NF-kB), leading to the activation of antiapoptotic and growth factor genes [9,12]. HHV-8 additionally produces interleukin-6 (IL-6) which, in addition to preventing apoptosis, was found to induce vascular endothelial growth factor (VEGF), increasing vascular permeability and augmenting the formation of PEL-related effusions. Immune evasion also plays a role, although this mechanism leaves much to be understood [9,12]. 

As in the case of our patient, the presentation usually begins with shortness of breath, the intensity of which depends on the location and the quantity of pleural effusion. It can also present with typical B symptoms such as weight loss, fever, and night sweats [9]. In rare cases, extra cavitary masses may also be found, typically in lymph nodes or adjacent to the body cavities [13]. Diagnostic thoracic or abdominal fluid tapping is often the next step to characterize the nature of effusion into exudative vs transudative type. PEL shows morphologic variability, which may include large immunoblastic, plasmablastic, or anaplastic B cell-lymphoma [14]. The cells are typically large, with moderate to abundant deeply basophilic cytoplasm, vacuoles, large round and irregular nuclei, and prominent nucleoli [7,9]. The confirmation of PEL relies on the detection of HHV-8 in the nuclei of malignant cells, with LANA protein detected on immunohistochemical stains [7,9,15]. They are described to have a “null” lymphocyte phenotype, as they do not exhibit typical B-cell or T-cell immunotype characteristics, although they exhibit CD45 in 93% of cases, proving that they are of lymphoid origin [7,9]. Oftentimes, the cells show positive or variable straining for plasma-associated markers CD138, CD38, IRF4/ MUM1, and EMA [7,15]. CD30 is often positive in 73% of the cases [7]. Neoplastic cells often lack expression of the B-cell markers CD19, CD20, CD79a, and PAX5, and surface and cytoplasmic immunoglobulins are usually negative or minimally expressed [7,15]. Occasionally, CD3 may be seen, although T-cell and NK-cell antigens are generally negative [7,15]. Some cases also show a BCL6 mutation [15].

While parapneumonic effusion due to coexisting bacterial pneumonia was a differential in our case, a lymphocyte-predominant pleural fluid analysis, negative pleural fluid culture reports, and morphologic and immunophenotypic evidence helped narrow the diagnosis to PEL. Our case had the typical picture of HHV-8 positivity, along with CD45, CD43, and CD30 on immunophenotyping and morphologic characteristics of PEL on microscopy. Furthermore, other differentials like systemic lymphomas with secondary involvement of body fluids, extranodal Burkitt lymphoma, pyothorax associated lymphoma were unlikely with HHV-8 positive pleural fluid analysis, negative HIV status, pleural fluid negative for AFB smear and culture, etc. Lastly, PET-CT findings reporting no evidence of recurrent disease in GIT or hypermetabolic activity ruled out malignant effusion as a consequence of the recurrence of underlying gastric adenocarcinoma. 

Due to the rarity of the condition, prospective clinical trials to guide treatment regimens for PEL are lacking. As in other aggressive lymphomas, chemotherapy is the main modality of treatment. It includes dose-adjusted etoposide, prednisolone, vincristine, cyclophosphamide, doxorubicin regimen (EPOCH) or cyclophosphamide, doxorubicin, vincristine, and prednisone (CHOP) regimen [16]. Patients with PEL with underlying HIV are managed with antiretroviral therapy (ART) and those with significant fluid deposition in body cavities are managed with therapeutic fluid removal for symptomatic relief. CHOP regimen in HIV-associated PEL has been shown to induce remission in a single institution study involving 11 patients with PEL [17]. In contrast, both large- and small-scale studies regarding appropriate chemotherapy regimens in HIV-negative immunocompetent patients with PEL are lacking. Chemotherapy regimens are often formulated by extrapolating the available evidence from HIV-positive PEL cases. Guler and colleagues have reported non-response to the CHOP regimen in the EBV-positive solid variant of PEL [18]. On the other hand, PEL with complete remission on a tapering dose of steroid therapy alone is also reported in an 84-year-old HIV-negative patient with PEL [19]. The prognosis is generally poor, with an average survival time of less than 24 months, despite treatment with both antiretrovirals and chemotherapy [20]. In a study by Narkhede and colleagues involving 104 patients with PEL, the median survival rate was found to decrease from 18 months to four months in patients with single vs multiple cavity involvement [9]. Early detection and prompt management, therefore, remains imperative in these cases.

## Conclusions

Primary effusion lymphoma (PEL) is a rare, aggressive, high-grade type of non-Hodgkin’s lymphoma (NHL) often associated with immunosuppression. The prognosis of the condition is gravely affected by delayed diagnosis. Although an increasing number of cases are associated with immunosuppression, a large number of cases are diagnosed in immunocompetent individuals as well. Despite its aggressive nature, PEL may present as a simple pleural effusion that can be easily attributed to other clinical entities such as pneumonia, infections, or heart failure, which can dangerously lead to a missed diagnosis. Therefore, a high index of suspicion must always be observed for early detection of the disease. Lastly, pleural fluid analysis should be repeated in patients with a high index of suspicion to prevent missing out on false negative reporting of pleural fluid analysis as in our case.
